# Factors contributing to the high prevalence of multidrug-resistance/Rifampicin-resistance in patients with tuberculosis: an epidemiological cross sectional and qualitative study from Khabarovsk krai region of Russia

**DOI:** 10.1186/s12879-022-07598-7

**Published:** 2022-07-13

**Authors:** Ilia Bykov, Olga Dyachenko, Pavel Ratmanov, Huan Liu, Libo Liang, Qunhong Wu

**Affiliations:** 1 Health Management College, Social Medicine Department, Harbin Medical University, 157 Baojian Road, Nangang District, Harbin, Heilongjiang 10081 People’s Republic of China; 2 Public Health and Health Care Department, Far Eastern State Medical University, Khabarovsk, Russia; 3 Internal Diseases Department with the Course of Phthisiology, Far Eastern State Medical University, Khabarovsk, Russia

**Keywords:** Multidrug-resistant/Rifampicin-resistant tuberculosis, Risk factors, Tuberculosis, Disability, Penitentiary facilities

## Abstract

**Background:**

Growing prevalence of multidrug-resistant/Rifampicin-resistant tuberculosis (MDR/RR-TB; resistance to Isoniazid and Rifampicin/Isolated resistance to Rifampicin) is putting in jeopardy the WHO End TB strategy. This study aimed to identify factors contributing to the high prevalence of MDR/RR-TB in Khabarovsk krai region of Russia.

**Methods:**

A cross-sectional retrospective study was conducted, analyzing clinical, demographic, and drug susceptibility testing data on 1440 patients. As a source of raw data, the national electronic TB surveillance system was used. Anonymous data was collected on every patient diagnosed with TB in all healthcare facilities of the region from January 2018 to December 2019. Only patients with proven excretion of *m. tuberculosis* were included in the study. Factors associated with MDR/RR-TB were identified through logistic regression analysis, in conjunction with in-depth interviews with eight patients, five healthcare managers and five doctors.

**Findings:**

2661 patients were identified with TB, 1440 were incorporated in the study based on inclusion criteria. Of these, 618 (42.9%) were identified with MDR/RR-TB. Patients with a history of imprisonment were 16.53 times (95% CI 5.37 to 50.88,) more likely to have MDR/RR-TB, whereas re-treatment patients were 2.82 times (95% CI 2.16 to 3.66) more likely to have MDR/RR-TB. Other influencing factors included presence of disability (AOR is 2.32, 95% CI 1.38 to 3.89), cavitary disease (AOR is 1.76, 95% CI 1.37 to 2.25), and retirement status (AOR 0.65, 95% CI 0.43 to 0.98, p = 0.042). Poor patient knowledge and understanding of the disease, progressive weariness of prolonged TB treatment, and inability hospitalize infectious patients without their consent were perceived by the interviewees as major influencing factors.

**Conclusions:**

Incarceration and treatment history, regardless of outcome, were identified as major factors influencing MDR/RR-TB prevalence. It is essential for the TB care system to eliminate legal loopholes, which deprive doctors of means to enforce quarantine procedures and epidemiological surveillance on infected patients, former and current inmates. Increasing people’s awareness of TB, early detection and appropriate treatment of patients with TB are needed for successfully combating MDR/RR-TB.

## Introduction

The continuous increase of multidrug-resistant/Rifampicin-resistant tuberculosis (MDR/RR-TB; resistance to Isoniazid and Rifampicin/Isolated resistance to Rifampicin) throughout the globe is a major public health issue. In 2020, the WHO reported that the global treatment success rate for MDR/RR-TB was only 59%. Treatment of MDR/RR-TB is more toxic to the patient, less effective, and it carries a much higher economic burden on the patients and the healthcare system, jeopardizing TB control efforts in developed and developing countries alike [1–4].

Russia carries one of the greatest burdens of MDR/RR-TB in the world. In 2018, MDR/RR-TB accounted for 35% of all new TB cases and 71% of all previously treated TB cases, with an MDR/RR-TB incidence of 27 per 100,000 and second highest in the world MDR/RR-TB prevalence rate [5, 6].

**Fig. 1:**
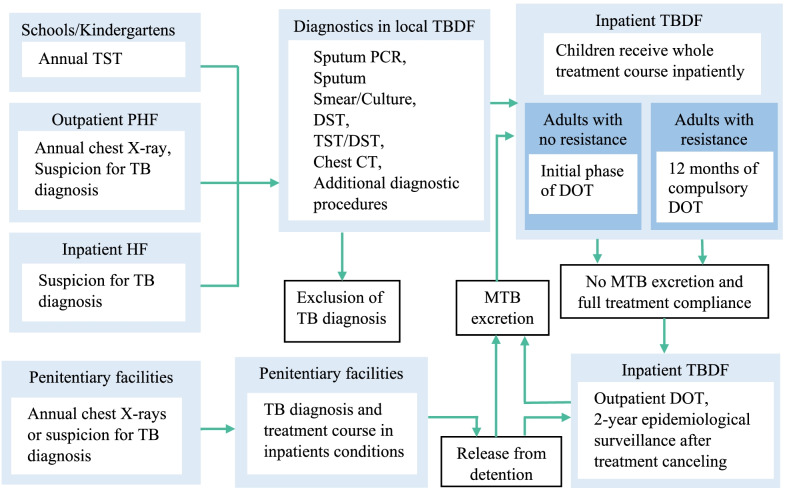
Organization of TB care in Russia. *PHF* primary healthcare facility, *HF* healthcare facility, *TBDF* TB designated facility, *DOT* directly observed treatment, *PCR* polymerase chain reaction, *TST* tuberculin skin test, *DST* drug susceptibility testing, *DST* Diaskin skin test, *CT* computed tomography, *MTB* mycobacterium tuberculosis

Such situation develops despite what seems to be a well-designed TB care system, in which all aspects of TB care in Russia are free of charge to the public. The entire system was designed to provide universal, quality, and specialized care that is centralized in TB-designated facilities (TBDF), as depicted in Fig. 1. All healthcare facilities and schools are participating in detection of asymptomatic TB by performing a mandatory annual chest x-ray in adults and Tuberculin Skin Testing (TST) in children. If TB is suspected, the healthcare facility is legally obliged to transfer such a patient to the TBDF, so the final diagnosis of suspected TB patients and provision of TB treatment are reserved only for TBDF [7]. The only exception to this system are penitentiary facilities, which screen, diagnose and treat inmates themselves. Such independence has been already proposed by researchers as a main reason for the high incidence of TB in Russian prisons, but little has been done to improve this situation [8].

High MDR/RR-TB incidence encourages the government of the Russian Federation and the Russian society of Phthisiatricians^1^ have developed specific policies and guidelines to address the issues [9]. Joint TB control programs with international partners have been initiated, research funds have been extended, a centralized national electronic database of TB patients created, and new diagnostic techniques and policies implemented. However, the progress has been far from satisfactory, as despite the decrease in total TB incidence, the prevalence of MDR/RR-TB is continuing to rise [5–8].

It is crucial to identify the underlying reasons for such high MDR/RR-TB prevalence so that more feasible and cost-effective approaches can be developed to control this trend. International reviews, and some country-level studies, have identified that risk factors for MDR/RR-TB vary depending on country, region, or locality. Among the most prevalent are poor adherence to TB treatment on the part of patients, poor monitoring and management of TB treatment adverse events, poverty, overcrowding, HIV co-infection, diabetes, alcoholism, smoking, cavitary disease and young age [10–18].

This study was undertaken in Khabarovsk Krai, in the Far East of Russia, where the proportion of MDR-TB among newly diagnosed cases (38%) was estimated to be the third highest in the country, significantly higher than the national average of 29.3% in 2018 [19]. Unfortunately, there is a notable gap in research into MDR/RR-TB rates between high and low prevalence regions of Russia. Despite some studies carried out in low-prevalence regions, factors influencing MDR/RR-TB in high- prevalence regions have rarely been investigated [20–22].

## Methods

### Data collection

Khabarovsk Krai is a region in the Far East of the Russian Federation, and it considered one of its high-prevalence TB regions. The TB incidence there is 87.7 per 100,000 in 2019 (almost twice the national average) with a population of only 1.2 million. These figures relegate region to ninth place by total TB incidence out of all 85 regions of Russia [19].

The mixed epidemiological, cross-sectional, retrospective and a qualitative study was conducted in the central “Tuberculosis Hospital” of Khabarovsk Krai. This institution collects and processes information about all patients with tuberculosis in region and functions as a reference laboratory at the regional level.

The Federal Register of TB patients (FRTP) was used as a source of data, which is a subsystem of the digital Unified State Healthcare Information System (USHIS). FRTP stores data of TB patients in form of electronic medical records, which were accessed by the investigator in April 2021. Anonymous demographic and clinical characteristics of every patient that was diagnosed with TB from January 2018 to December 2019 was collected and summarized into single data set for further analysis. The surveillance covered all healthcare institutions in the region and strictly followed the guidelines developed by the WHO and the International Union against Tuberculosis and Lung Disease [23, 24].

### Diagnostic methods

In all patients with suspected TB in the region, an acid-fast bacilli sputum smear, culture (with both liquid and solid media), real-time PCR, and Drug Susceptibility Testing (DST) are routinely implemented as initial diagnostic tests.

Various DST techniques were employed to detect MDR/RR-TB in patients. A liquid and solid media sputum culture tested MTB resistance to the following agents: Isoniazid, Rifampicin, Ethambutol, Streptomycin, Linezolid, Capreomycin, Amikacin, Ofloxacin, Levofloxacin, Kanamycin, Ethionamide, Para-aminosalicylic acid and Cycloserine. DST in direct PCR was performed with Allele-specific polymerase chain reaction aimed to identify rpoB, katG, inhA, embB and gyrA genes mutations associated with resistance to Isoniazid, Rifampicin, Ethambutol and fluoroquinolones [25]. All DST strictly followed the procedures and methods set out by WHO and the International Union against Tuberculosis and Lung Disease [24].

A new patient was defined as a patient with TB who had never been treated for TB previously, or who had received anti-TB drugs treatment for less than 1 month. A re-treatment patient was defined as a patient who had received anti-TB drug treatment for more than 1 month [10].

### Inclusion/Exclusion criteria

A patient whose sample tests positive for mycobacterium tuberculosis (MTB) in at least one of the four initial diagnostic tests was defined as having active pulmonary TB. Patients whose samples are negative for the MTB in all four initial diagnostic tests were defined as having non-active pulmonary TB. Such patients were excluded from the study. Patients with mono-resistance (resistance to one first-line anti-TB drug only), poly-resistance (resistance to more than one first-line anti-TB drug, other than both isoniazid and rifampicin) and extensive drug resistance (resistance to any fluoroquinolone, and at least one second-line injectable TB medication, in addition to multidrug resistance) were excluded from the study. Patients whose electronic medical records contained incomplete set of data were also excluded from the study.

### In-depth interviews

The interviewees were purposely selected based on their roles and experience in TB control. A total of 8 re-treatment and newly diagnosed patients completed the interviews, including one with a history of incarceration. 10 healthcare workers involved with their treatment completed interviews, including 5 physicians and 5 health administrative officials. Patients with TB were asked to answer when, where, and why they had received TB services, how they were treated and what problems they faced. The administrative officials were asked to answer questions about the arrangement of treatment regimens and compliance of providers and patients. The physicians were asked to answer questions about their experiences and problems in treating TB patients. The interviews were digitally recorded, transcribed, and thematically coded. The final sample size was determined by a saturation of information when no new categories emerged*.*

### Quantitative data analysis

Quantitative variables of demographic and clinical characteristics of the patients with MDR/RR-TB were compared with those without MDR/RR-TB. Three regression models were constructed for all cases combined—only new cases and only re-treatment cases, respectively. Tested in the regression analysis were the following independent variables: gender, age, social status, place of residence, living conditions, substances abuse, a history of imprisonment, partial or total permanent inability to work (further—disability), HIV status, a history of treatment with Rifampicin and Isoniazid, TB localization, cavitary disease, and circumstances of TB discovery. For the re-treatment patients, additional variables of previous therapy outcome and length of previous treatment with Rifampicin and Isoniazid were added.

Three separate binary logistic regression models were created. Chi square tests were used for testing the statistical significance of the models as a whole. Fitness of the models to the data were tested with the Hosmer & Lemeshow test. Regression coefficient, Odds ratio with 95% confidence interval (95% CI) for each variable category was calculated and Wald statistics method used to determine their statistical significance [26]. An analysis was performed using SPSS software V24.0.01.

### Qualitative data analysis

The interview data was analyzed thematically, with the coding framework developed inductively from the data. The initial coding used open coding and theoretical coding. The initial codes were then refined to produce a smaller set of themes. The coding framework was subject to continuing revision with each iteration during analysis. Main objectives of the analysis were to receive clinical explanation for the results of quantitative data analysis, identify non-apparent factors influencing MDR/RR-TB incidence, determine features of the TB care system organization and limitations of the study. An analysis was performed using Nvivo software V11.0.

## Results

### Characteristics of the study population

**Fig. 2:**
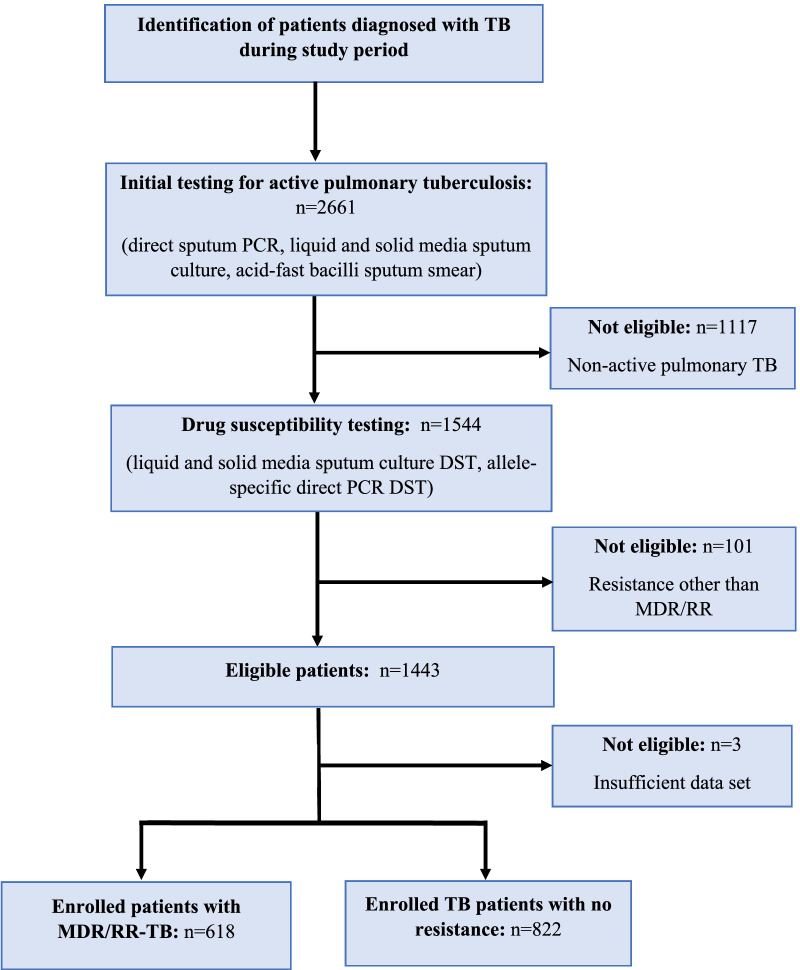
Flow chart of patients enrollment. *PCR* polymerase chain reaction, *DST* drug susceptibility testing, *MDR/RR-TB* multidrug-resistant/Rifampicin-resistant tuberculosis

During the study period, in all healthcare institutions of the region, 2661 patients were diagnosed with TB, of which 1544 patients had an active pulmonary tuberculosis, and 101 patients were excluded from the study due to resistance types other then MDR/RR-TB, three patients were excluded due to an incomplete set of variables leaving 1440 patients for further analysis (Fig. 2).

Participants had a median age of 41 years, and 73.5% of them were male. MDR/RR was detected in 618 participants (42.9%, 95% CI 40.3% to 45.5%). The proportion of participants residing in cities was 69.4%, and 989 of them (68.7%) had no record of employment. By residence type, 61.5% were living in separate apartments and 151 (10.5%) were homeless at the time of TB diagnosis. There were 2.01 times more homeless among re-treatment cases than in newly diagnosed cases (16,5% vs 8.2%). Alcoholism was reported in 3.5% of all cases and in 62.5% of TB was discovered actively with an annual chest x-ray in primary healthcare facilities (PHF). More than half of all cases (55.1%) showed signs of cavitary disease. Disabilities were reported in 6% of the entire sample, while 8.3% were tested positive for HIV. 78 patients (5.4%) were in detention or had an official history of incarceration at the time of diagnosis. Overall, incarcerated patients had a much higher prevalence MDR/RR (93.6%, 95% CI 85.6% to 97.8%) compared to the population without a history of imprisonment (40.0%, 95% CI 37.4% to 42.7%) (p < 0.001).

Out of the 1440 patients, 394 were re-treatment patients, totaling 27.4% of the overall sample. The re-treatment patients had greater prevalence of MDR/RR (64.6%, 95% CI 59.8% to 69.4%) than the new patients (34.8%, 95% CI 31.8% to 37.7%) (p < 0.001) (Table 1).

**Table 1:** Demographical and clinical characteristics of the recruited patients:

Variable	Entire sample N and % (n = 1440)	New cases N and % (n = 1046)	Retreatment cases N and % (n = 394)	MDR/RR/Non-MDR/RR-TB (p value)
MDR/RR				
MDR/RR	618 (42.9)	363 (34.7)	255 (64.7)	(in applicable)
No MDR/RR	822 (57.1)	683 (65.3)	139 (35.3)	
Gender				
Male	1058 (73.5)	755 (72.2)	303 (76.9)	0.856
Female	382 (26.5)	291 (27.8)	91 (23.1)	
Age, median years (range)				
< 41 years	715 (43.7)	524 (50.1)	191 (48.5)	< 0.001
≥41 years	725 (50.3)	522 (49.9)	203 (51.5)	
Place of residence				
Country side	440 (30.6)	311 (29.7)	129 (32.7)	0.149
City	1000 (69.4)	735 (70.3)	265 (67.3)	
Social status				
Not working	989 (68.7)	675 (64.5)	314 (79.7)	(inapplicable)
Working	244 (16.9)	203 (19.4)	41 (10.4)	
Retired	173 (12.0)	136 (13.0)	37 (9.4)	
Student	28 (1.9)	26 (2.5)	2 (0.5)	
Not organized child	4 (0.3)	4 (0.4)	0	
In military service	2 (0.1)	2 (0.2)	0	
Living conditions:				
Homeless	151 (10.5)	86 (8.2)	65 (16.5)	(inapplicable)
Bed in designated facility	44 (3.1)	12 (1.1)	32 (8.1)	
Room in Dormitory	13 (0.9)	9 (0.9)	4 (1.0)	
Room in separate apartment	11 (0.8)	10 (1.0)	1 (0.3)	
Separate house	335 (23.3)	241 (23.0)	94 (23.9)	
Substances abuse				
No official records	1385 (96.2)	1006 (97.8)	379 (96.2)	(inapplicable)
Alcoholism	51 (3.5)	37 (3.5)	14 (3.6)	
Drug abuse	4 (0.3)	3 (0.3)	1 (0.3)	
Circumstance of TB discovery				
Visit with symptoms to healthcare facility	540 (37.5)	422 (40.3)	118 (29.9)	0.005
Discovered actively by healthcare facility	900 (62.5)	624 (59.7)	276 (70.1)	
Imprisonment history				
No official history of detention	1362 (94.6)	1023 (97.8)	339 (86.0)	< 0.001
Being in detention or history of detention	78 (5.4)	23 (2.2)	55 (14.0)	
Cavitary disease				
No cavities	646 (44.9)	483 (46.2)	163 (41.4)	< 0.001
Presence of cavities	794 (55.1)	563 (53.8)	231 (58.6)	
Disabilities				
No disability	1354 (94.0)	1001 (95.7)	353 (89.6)	< 0.001
Presence of disability	86 (6.0)	45 (4.3)	41 (10.4)	
TB localization:				
Pulmonary TB	1401 (97.3)	1011 (96.7)	390 (99.0)	(inapplicable)
TB of lungs’ lymph system, pleural	29 (2.0)	26 (2.5)	3 (0.8)	
Non thoracic TB	10 (0.7)	9 (0.9)	1 (0.2)	
HIV status				
Negative	1321 (91.7)	967 (92.4)	354 (89.8)	< 0.001
Positive	119 (8.3)	79 (7.6)	40 (10.2)	

*HIV* human immunodeficiency virus, *TB* tuberculosis, *MDR/RR* multidrug resistance/Rifampin resistance

### Factors associated with MDR/RR-TB

Data of 1440 patients undergone logistic regression analysis. The strongest associative factors for MDR/RR-TB were a history of imprisonment and history of previous treatment. In these cases, the risk of having MDR/RR-TB was 16.53 (95% CI 5.37 to 50.88, p < 0.001) and 2.82 times higher (95% CI 2.16 to 3.66, p < 0.001) respectively.

Other influencing factors included presence of disability (AOR is 2.32, 95% CI 1.38 to 3.89, p = 0.001), cavitary disease (AOR is 1.76, 95% CI 1.37 to 2.25, p < 0.001), positive HIV status (AOR 1.55, 95% CI 1.01 to 2.39, p = 0.046), age (AOR for being older than 41 years is 1.36, 95% CI 1.06 to 1.76, p = 0.013), place of residence (AOR for residing in the city is 1.44, 95% CI 1.09 to 1.89, p = 0.01). The only variable that showed protective effect against MDR/RR-TB was retirement status (AOR 0.65, 95% CI 0.43 to 0.98, p = 0.042) (Table 2).

**Table 2:** Analysis for estimation of prevalence of MDR/RR-TB in the entire sample (n = 1440)

Variable	Prevalence of MDR/RR-TB (%)	AOR (95% CI)	P value
Gender			
Female	43.1	1.21 (0.93 to 1.57)	0.156
Male	41.4	1 (reference)	
Age, median years (range)			
≥41 years	48	1.36 (1.06 to 1.76)	0.013
< 41 years	37.9	1 (reference)	
Place of residence			
City	44.2	1.43 (1.09 to 1.89)	0.010
Countryside	40.0	1 (reference)	
Social status (whole variable)		(–)	
Working	39.3	0.75 (0.09 to 6.53)	0.432
Retired	29.3	0.65 (0.43 to 0.98)	0.792
Student	35.7	0.94 (0.41 to 2.15)	0.042
Not organized child	50	1.08 (0.79 to 1.47)	0.879
In military service	100	–	0.639
Not working	46.3	1 (reference)	0.999
Living conditions (whole variable)		–	0.125
Bed in designated facility	51.3	0.98 (0.29 to 3.37)	0.608
Room in dormitory	86.4	(–)	0.656
Room in separate apartment	46.2	0.84 (0.45 to 1.56)	0.569
Separate house	9.1	2.35 (0.23 to 24.25)	0.475
Separate apartment	43.9	1 (reference)	-
Substances abuse (whole variable)		(–)	0.656
Alcoholism	39.2	0.84 (0.45 to 1.56)	0.569
Drug abuse	75	2.35 (0.23 to 24.25)	0.475
No official records	43	1 (reference)	-
Circumstance of TB discovery			
Discovered actively by healthcare facility	45.8	1.24 (0.97 to 1.60)	0.088
Visit with symptoms to healthcare facility	38.1	1(reference)	
Imprisonment history			
Being in detention or history of detention	93.6	16.53 (5.37 to 50.88)	< 0.001
No official history of detention	40.0	1 (reference)	
Cavitary disease			
Presence of cavities	47.4	1.76 (1.37 to 2.25)	< 0.001
No cavities	37.5	1 (reference)	
Disabilities			
Presence of disability	67.4	2.32 (1.38 to 3.89)	0.001
No disability	41.4	1 (reference)	
TB localization		(–)	0.859
Pulmonary TB	43.4	1.27 (0.55 to 2.94)	0.581
Non thoracic TB	0	–	–
TB of lungs’ lymph system, pleural	34.5	1 (reference)	0.999
HIV status			
Positive	53.8	1.55 (1.01 to 2.39)	0.046
Negative	41.9	1 (reference)	
Treatment history with I and R			
Retreatment	64.6	2.82 (2.16 to 3.66)	< 0.001
No history	34.8	1 (reference)	

*HIV* human immunodeficiency virus, *TB* tuberculosis, *MDR/RR* multidrug resistance/Rifampin resistance, *AOR* adjusted odds ratio

The multilevel model for the newly diagnosed patients was composed of 1047 cases. Of these, 364 had MDR/RR-TB (34.7%). History of imprisonment was the strongest associative factor, with 11.9 times higher risk of MDR/RR-TB (95% CI 2.94 to 43.78, p < 0.001).

Among remaining factors, three showed association with presence of MDR/RR-TB. Cases with underlying cavitary disease were 1.96 times more likely to have MDR/RR-TB (95% CI 1.46 to 2.63, p < 0.001). Patients living in cities were 1.841 times more likely to have MDR/RR-TB (95% CI 1.25 to 2.44, p = 0.001). Positive HIV status was associated with 1.67 times increase in MDR/RR-TB risk (95% CI 1.01 to 2.77, p = 0.047) (Table 3).

**Table 3:** Analysis for estimation of prevalence of MDR-TB in the new cases (n = 1046) and retreatment cases (n = 394)

Variable	New cases (n = 1046)			Retreatment cases (n = 394)		
	Prevalence of MDR-TB (%)	AOR (95% CI)	p value	Prevalence of MDR-TB (%)	AOR (95% CI)	p value
Gender						
Female	36.4	1.25 (0.92 to 1.69)	0.157	65.6	1.24 (0.71 to 2.18)	0.441
Male	34.1	1 (reference)	-	61.5	1 (reference)	–
Age, median years (range)						
≥41 years	39.5	1.34 (0.99 to 1.81)	0.013	58.1	0.65 (0.38 to 1.09)	0.108
< 41 years	30.0	1 (reference)	–	71.6	1 (reference)	–
Place of residence						
City	37.2	1.841 (1.32 to 2.57)	0.010	66.7	1.27 (0.72 to 2.26)	0.408
Countryside	28.9	1 (reference)	-	63.6	1 (reference)	–
Social status (whole variable)		(–)	0.604	–	(–)	0.008
Working	30.5	0,83 (0.58 to 1.19)	0.639	82.9	4,32 (0.58 to 1.19)	0.002
Retired	24.8	0.66 (0.41 to 1.07)	0.042	45.9	0.66 (0.29 to 1.49)	0.042
Student	30.8	0.75 (0.31 to 1.86)	0.879	100.0	–	0.999
Not organized child	50.0	0.81 (0.09 to 6.81)	0.792	0.0	–	0.999
In military service	100.0	–	0.999	0.0	–	0.999
Not working	37.9	1 (reference)	–	64.2	1 (reference)	–
Living conditions (whole variable)		(–)	0.534		(–)	0.526
Bed in designated facility	66.7	0.73 (0.12 to 4.37)	0.608	93.8	0.43 (0.05 to 3.68)	0.439
Room in dormitory	22.2	0.64 (0.12 to 3.43)	0.978	100.0	–	0.999
Room in separate apartment	0.0	–	0.999	100.0	–	0.999
Separate house	37.3	1.379 (0.79 to 2.40)	0.595	60.6	0.78 (0.35 to 1.77)	0.560
Separate apartment	33.7	0.98 (0.60 to 1.61)	0.28	59.1	0.52 (0.26 to 1.05)	0.070
Homeless	37.2	1 (reference)	–	70.3	1 (reference)	–
Substances abuse (whole variable)		(–)	0.991		(–)	0.539
Alcoholism	32.4	0.97 (0.47 to 1.99)	0.935	57.1	0.48 (0.13 to 1.73)	0.266
Drug abuse	66.7	1.15 (0.09 to 14.14)	0.914	100.0	–	0.999
No official records	34.8	1 (reference)	–	64.8	1 (reference)	–
Circumstance of TB discovery						
Discovered actively by healthcare facility	35.3	1.18 (0.88 to 1.57)	0.278	69.6	1.79 (1.06 to 3.05)	0.030
Visit with symptoms to healthcare facility	34.0	(reference)		53.0	1 (reference)	
Imprisonment history						
Being in detention or history of detention	82.6	11.9 (3.08 to 45.93)	< 0.001	98.2	38.55 (3.65 to 407.42)	0.002
No official history of detention	33.7	1 (reference)		59.2	1 (reference)	
Cavitary disease						
Presence of cavities	40.1	1.95 (1.45 to 2.62)	< 0.001	65.2	1.46 (0.87 to 2.44)	0.151
No cavities	28.6	1 (reference)		63.8	1 (reference)	
Disabilities						0.008
Presence of disability	48.9	1.66 (0.87 to 3.15)	0.119	87.8	4.44 (1.47 to 13.38)	
No disability	34.1	1 (reference)		61.9	1 (reference)	
TB localization:		(–)	0.494		(–)	0.999
TB of lungs’ lymph system, pleural	38.5	1.69 (0.71 to 4.04)	0.235	0.0	–	
Non thoracic TB	0.0	–	0.999	0.0	–	
Pulmonary TB	35.0	1 (reference)	–	65.3	1 (reference)	
HIV status						
Positive	46.8	1.67 (1.01 to 2.77)	0.047	67.5	1.48 (0.62 to 3.53)	0.378
Negative	33.8	1 (reference)		64.3	1 (reference)	–
Previous therapy						
More than 180 days	–	–	–	61.4	0.92 (0.56 to 1.51)	0.732
Less than 180 days				69.7	1 (reference)	
Previous therapy outcome (whole variable)						0.061
Not effective course	-	–	–	79.3	(–)	0.621
Other				0.0	1.379 (0.79 to 2.40)	0.999
Transferred				100.0	–	0.999
Interrupted course				52.5	–	0.089
Effective course				61.4	0.98 (0.60 to 1.61)	–

HIV human immunodeficiency virus, TB tuberculosis, MDR/RR multidrug resistance/Rifampin resistance, AOR adjusted odds ratio

393 re-treatment patients were analyzed in the separate multilevel model. Four variables proved to be significant, with a history of incarceration remaining the strongest associative factor. Such patients were 38.5 times more likely to have MDR/RR-TB (95% CI 3.64 to 407.42, p = 0.002). The presence of disability led to a 4.43 times greater likelihood of contracting MDR/RR-TB (95% CI 1.47 to 13.38, p = 0.008). A final two significant variables were official records of employment (AOR 4.32, 95% CI 1.74 to 10.71, p = 0.002) and being discovered actively by PHF (AOR 1.79, 95% CI 1.05 to 3.05, p = 0.03) (Table 3).

### Interpretation of interviews

Healthcare providers confirmed that former inmates are the most problematic group of TB patients. TB care in penitentiary facilities is provided on site, by the often undertrained medical stuff with insufficient funding and equipment. This medical service is not controlled by the authorities of general TB care system and operates without supervision. Lack of centralized control in tandem with poor nutrition and living conditions, overcrowding, treatment regimen neglect by prisons’ medical staff and inmates, make prisons the clearest breeding ground for drug resistance. After being released, prisoners mostly disappear from epidemiological surveillance and, even if they show up for further treatment, they often fail to adhere to the regimen and follow-up procedures and there are no legal tools for healthcare providers to enforce surveillance on them.

Among interviewees, there was a consensus that the main obstacle toward achieving full patient’s compliance is the length of therapy. Patients become progressively tired as the treatment advances, especially those with drug resistance, whose regimens typically last for 24 months or more.

In these cases, all patients with MDR/RR-TB receive treatment in inpatient conditions for at least twelve months in TBDF. After that period, the patient can be transferred to an outpatient treatment regimen. This option is reserved for patients who have proven themselves trustworthy, showing full compliance during inpatient treatment, and not actively secreting TB mycobacterium so as not to be an ongoing source of infection to others. Patients who do not meet those criteria continue their treatment in inpatient conditions until the end of the course. Such prolonged treatment, combined with insufficient awareness about consequences of intermittent treatment, leads patients to forgo treatment, usually as soon as symptoms disappear.

Outpatients phase of regimens provide many opportunities for the patients to drop out due to various reasons. Local TBDF in collaboration with the Russian Red Cross NGO are trying to address this issue by more thoroughly educating patients about TB treatment, giving out free monthly food packages and reimbursement of travel expenses to fully compliant patients. According to physicians, this has been especially effective in encouraging economically disadvantaged patients to continue their treatment.

Neither patients nor healthcare providers reported adverse effects to be a significant issue in achieving full treatment adherence. During an inpatient stay, patients are closely monitored for occurrence of adverse effects, where they are also given medication for side effects prevention. Physicians stated that they are equipped with all the necessary medications for the effective management of adverse effects.

Monitoring the occurrence of the adverse events among outpatients is carried out through mandatory monthly examination. The only issue here, that during ambulatory phase of the treatment, patients pay out-of-pocket for drugs to combat minor side effects, but in event of adverse effects occurrence during this phase physicians try to hospitalize such patients in TBDF so they could receive free medication and avoid financial burden. Same couldn’t be said about medical service in penitentiary facilities, according to patients, monitoring procedures are next to non-existing and medications for combating adverse events are scarce there. So, it is common for the inmates to discontinue medications as soon as adverse event occurs.

Both physicians and healthcare managers reported that TBDF of the region are sufficiently supplied with quality anti-TB medication and all the necessary diagnostic equipment.

Healthcare providers stated that both outcome and history of previous treatment contributed to the resistance development contrary to our results that mere fact of previous treatment history is increasing risk of resistance presence regardless of its outcome. Same goes for social status and living conditions, which, according to our statistical data, do not influence resistance development, but from a clinical standpoint, socially disadvantaged patients (particularly homeless ones) display a greater tendency to acquire MDR/RR TB. Another substantial problem that was not supported by data but reported by interviewees, is a greater frequency of alcohol and substances abuse. MDR/RR–TB prevalence in those groups is significantly higher than average.

Physicians did not report any association between presence of disability and risk of MDR/RR-TB development but they stated that some patients purposely do not comply with treatment to worsen their condition and acquire disability status for receiving social security benefits.

## Discussion

Besides the TB mycobacterium’s major biological features leading to the prevalence of MDR/RR-TB, such as mutation potential [27], strains [28] and draft genome sequences [29], TB related service patterns, demographics and clinical factors also have a significant impact on the development of MDR/RR-TB. Based on the statistical analysis of 1440 TB patients, we identified several factors influencing incidence of MDR/RR-TB in the Khabarovsk region of Russia. Among them are incarceration and previous anti TB treatment history, cavitary disease, HIV co-infection, age, place of residence, retirement status, presence of disability, circumstance of TB discovery and social status. We also concluded that newly diagnosed patients and retreatment patients tend to have different risk factors for contracting and developing MDR/RR-TB. Qualitative survey showed that organization of the TB care system, features of TB treatment process, healthcare and social security legislation play important role in TB epidemiology and may influence incidence of MDR/RR-TB both directly and indirectly.

Findings of quantitative data analysis were partially supported by interviews with patients, health managers, and physicians. Incarceration history was identified as the strongest risk factor associated with MDR/RR-TB in all studied groups. In this data, a high prevalence of TB and its drug resistant forms can be seen in prisons all around the world, developed and developing countries alike [30–32].

In 2021, according to Birkbeck University of London, Russia occupied 5th place in the world in terms of total prison population [33]. Based on literature reports and the interpretation of interviews, we can conclude that persistent unhygienic incarceration conditions and low quality of medical care in penitentiary facilities cause high prevalence of MDR/RR among TB infected inmates [7]. This creates a substantial pool of hosts, carrying drug resistant TB in the general population. Contrary to all other healthcare facilities in Russia, penitentiary institutions’ medical service operates independently from the general TB care system (Fig. 1). Such demarcation deprives inmates of proper care and follow up, which they can receive in TBDF. Either a considerable reduction in the total prison population and/or inclusion of penitentiary facilities in the TB care system may address the problem.

This predicament of prisons is explained by a contradiction of Russian laws in the field of healthcare. No medical procedures can be instituted without a patient's consent, including hospitalization, so some patients simply leave TBDF at will. To ensure epidemiological surveillance, healthcare providers have to file a claim for forced hospitalization to the court, but there are no legal mechanisms to execute positive court decisions without a patient's written consent. Thereby, infectious TB patients can leave TBDF even with a court decision of forced hospitalization. In 2011 it was that 3500 out of 6000 lawsuits were satisfied in favor of forced hospitalization, and it took an average of 3 months to obtain a court decision and up to 30% of patients left TBDF after forced hospitalization [34].

To ensure proper TB surveillance, measures should be taken to eliminate such a loophole in the legal framework. For reducing the pool of former inmates with MDR/RR-TB, better treatment conditions and epidemiological surveillance in penitentiary facilities must be established. Such intervention of providing TB care for inmates in civil clinics has been implemented in the Tomsk oblast region of Russia from 2000 to 2002 [7], which consisted of two phases of DOTS-plus individualized MDR-TB treatment regimens. In first “intensive” inpatient phase, patients received a high calories and protein rich diet, a separate and better accommodation, and were closely monitored for occurrence of treatment’s side effects. In the second “continuation” ambulatory phase, a community-based approach was used to provide directly observed therapy in outpatient clinics. For avoiding treatment discontinuation, adverse effects were managed aggressively, alongside with provision of monthly food packages to fully compliant patients and reimbursement of travel expenses.

The results showed significant increase in compliance, reduction in adverse effects, decrease in mortality rate, and increased treatment efficiency among incarcerated patients. Listed interventions, contrary to penitentiary facilities, are common practice in civil TB designated clinics therefore success of this study supports the necessity of centralized TB care for all TB patients [8].

As it was reported by numerous studies, poor adherence to drug susceptible TB treatment and poor screening for MDR/RR-TB are the major threats to MDR/RR-TB spread. According to the WHO, in 2020 DST underwent 92% and 94% of all newly diagnosed and retreatment TB cases respectively [4]. Such large-scale testing combined with routine usage of direct PCR DST and culture-based DST methods in all TB suspected patients provide a reliable and effective system of monitoring and detection of MDR/RR-TB. This conclusion is supported by the results of healthcare providers’ interviews.

A much larger concern is the problem of the poor adherence to the TB treatment. Implementation of DOT and DOTS plus were beneficial but didn’t fully address this problem in Russia [7, 34]. As was reported, the length of therapy seems to be a major factor affecting a patient’s drop-out rate. Because of Russian legislation, it is challenging to keep patients in hospitals against their will but attempts to overcome this obstacle are present. For example, a special mobile medical team may visit some infected patients daily in their homes to deliver DOT. Those patients are typically not from economically disadvantaged groups, and their living conditions are much better than those of inpatient TB designated facilities, so their compliance is much higher when they stay in conditions that can be compared to home confinement but not in inpatient facility. Another instance is that pediatric TBDF provides compulsory school education to children, so they don’t disrupt their studies during a hospital stay.

A study from Belarus performed in 2009 identified positive association between disability and MDR/RR-TB prevalence [35]. Concerning another type of social security policies, influence of retirement social status on the risk of MDR/RR-TB development is uncertain as both positive and negative relationships were reported in the literature [13, 36]. A study conducted in Ethiopia revealed a tenfold increase in risk of MDR-TB for the military pensioners, explaining it as pensioners receive much fewer social benefits compared to the active military servicemen [13]. At the same time, researchers from Belarus describe a protective effect similar to results received in our study with a reported AOR of 0.6 [36]. Such consistency in results may be due to that Russia and Belarus, both being part former Soviet Union members, have similar social security policies regarding retirement benefits and benefits for people with permanent inability to work. There is a need for further investigation of that phenomenon in countries with and without such policies. Such studies may support disability status as a risk factor or reveal its connection to social security policies. It should be mentioned as limitation, that we had no data on whether disability was TB-related or determined by another disease, thus the full statistical meaning of this variable remains uncertain.

Our results concerning association between MDR/RR-TB with younger age, cavitary disease, and positive HIV status correspond with previous studies [28, 37, 38].

Regarding place of residency, city dwellers have better access to healthcare in general and have a far greater capacity to complete annual chest X-rays. Therefore, cities tend to see greater number of people diagnosed with TB than in rural areas. Likewise, the increased population density in cities provides more chances to contract MDR/RR–TB. This can explain an emerging trend observed by physicians—the increase in the prevalence of MDR / RR-TB among newly diagnosed patients.

Our study has several limitations. We failed to explain the significance of official employment status as a risk factor among re-treatment patients and insignificance of social status and living conditions, as these results contradict with previous studies [12, 38–40]. Also, our data indicates that outcome of previous treatment does not influence risk of MDR/RR-TB development which contradicts with previous studies and healthcare providers’ interviews [38]. During patients selection process, gyrB mutation of *m.tuberculosis* in DST was not tested due to usage of premanufactured reagent in allele-specific PCR [25], but because of simultaneous use of cultural DST methods we are confident that no patient with fluoroquinolones resistance was enrolled in the study.

It should be stated that data on narcotics and alcohol use disorders was based on official records of seeking medical attention for such conditions. Because significantly few patients in Russia seek medical attention for addiction-related problems [41] based on this and interviewee reports of high prevalence of these conditions in TB patients and corresponding literature [19], we acknowledge that our data may not fully represent the overall prevalence of substance abuse in the studied population.

It is worth mentioning that, even so according to the healthcare professionals’ interviews, TBDF is sufficiently supplied with medications of proper quality, our quantitative data didn’t contain information about supply and quality of anti-TB drugs. Another piece of valuable information that has not been represented in the quantitative data set was the occurrence of adverse effects. But based on the in-depth interview results, we can conclude that adverse events don’t affect compliance of patients receiving TB care in TBDF. Contrary to the penitentiary institutions where treatment drop-out due to adverse events occurrence is a major problem [31].

## Conclusion

Incarceration and treatment history, regardless of outcome, seems to be a major factor influencing MDR/RR-TB prevalence in Russia. It is essential for the TB care system to eliminate legal loopholes, which deprive doctors of means to enforce quarantine procedures and epidemiological surveillance on infected patients, former and current inmates, who seem to be the main source of MDR/RR-TB hosts in the general population. The last can be addressed by treating inmates in the facilities of the general TB care system. In the end, we want to say that further attempts to better patients’ education and public awareness about TB, with continuing coordinated and centralized care for patients with TB, in tandem with greater compliance by patients with TB treatment guidelines, are the keys to success in the battle against MDR/RR-TB globally.

## Data Availability

The datasets analyzed during the current study are under jurisdiction of corresponding authority body and are not publicly available but are available from the corresponding author on reasonable request due to need of corresponding authority body notification of data sharing with the third party.

## Footnotes

1 Phthisiatrician—a medical specialist trained in the diagnosis and treatment of tuberculosis. In Russia, historically, it stands out as a separate medical specialization.
